# Supplementary material for: Modeling Study on Optimizing Water and Nitrogen Management for Barley in Marginal Soils
Source: Plants (Basel). 2025 Feb 25;14(5):704. doi: 10.3390/plants14050704 (PMC11901529; doi:10.3390/plants14050704)
Supplement: Supplementary file 1 [file plants-14-00704-s001.zip › plants-3474992-supplementary.pdf]

Supplementary Materials:

Supplementary Figure S1. Soil profile and its main horizons.

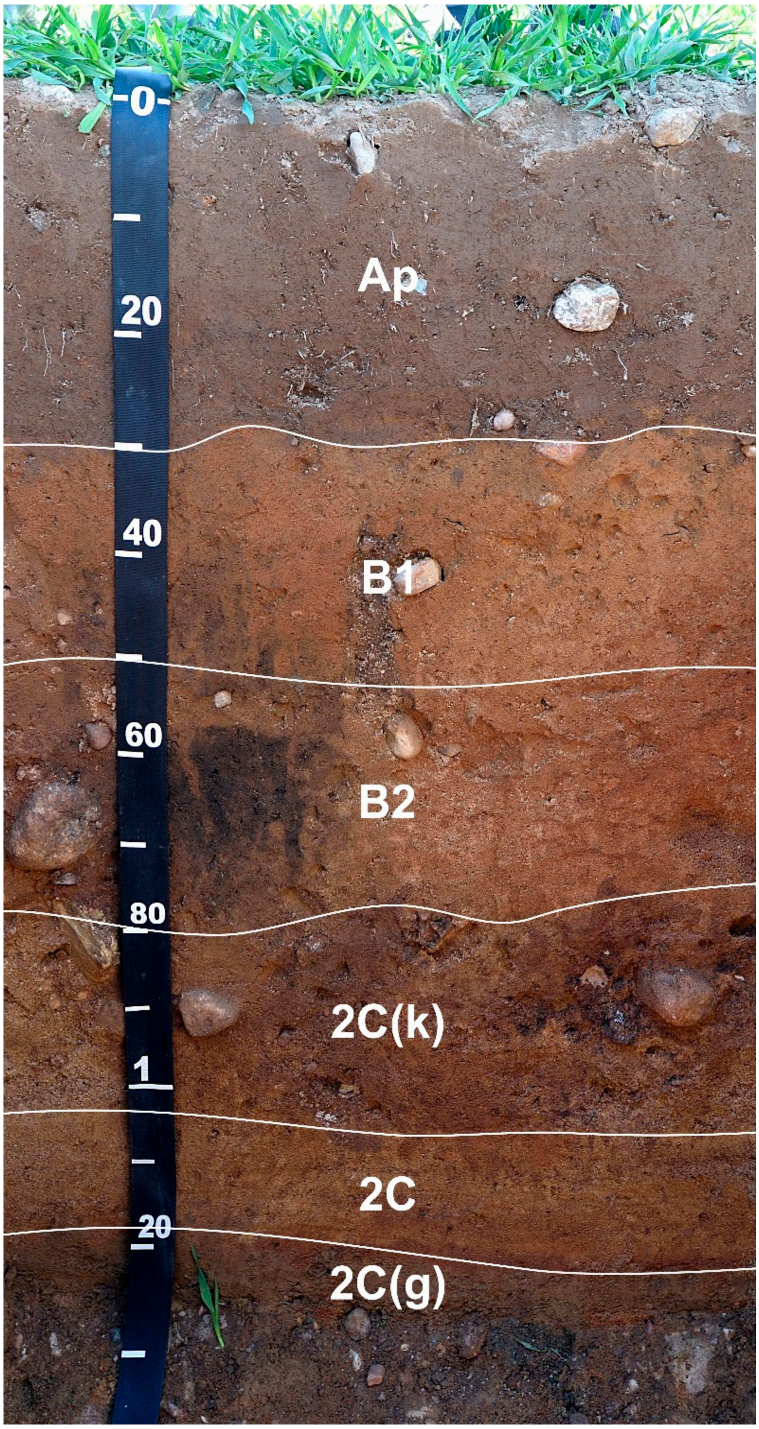

Supplementary Table S1 overview of the calibration steps and the calibrated parameters for the AgroC model.

Calibration step	calibration		Target	parameter	meaning	start parameters	bounds	fitted
	automatic	manual						
1	X		phenology	Tempstart	start temperature sum for growth	200	50-500	50.34
2	X		SWC dynamics	Ks of all layers and Theta_s for the last 3 layers	saturated hydraulic conductivity of the soil horizons	29; 10; 10; 10; 1.9; 0.285; 0.323; 0.312	40-60; 5-15; 5-15; 5-15; 1.8-2.0, 0.26-0.32, 0.28-0.34, 0.28-0.34	59.872; 9.3423; 5.8785; 5.054; 1.9496; 0.31466; 0.33801; 0.28028
3		X	photosynthesis rate (biomass production)	Vc_{max},; Ball/Berry *m*	Farquar parameters for light use efficiency	------	------	36; 7
4		X	nitrate uptake by plants	rdo; Kd	travel distance resistance between bulk soil and root; ammonium sorption constant per layer	------	------	50000; 879.5; 87.4; 87.4; 87.4
